# Plasma vascular non-inflammatory molecule 3 is associated with gastrointestinal acute graft-versus-host disease in mice

**DOI:** 10.1186/s12950-017-0178-z

**Published:** 2018-01-05

**Authors:** Na Wang, Xiaoyi Qin, Yigeng Cao, Bin Liang, Kang Yu, Haige Ye

**Affiliations:** 1 Wenzhou Medical University, Wenzhou, Zhejiang 325002 China; 2 Institute of Hematology and Blood Diseases Hospital, Chinese Academy of Medical Sciences and Peking Union Medical College, Tianjin, 300020 China; 3 Department of Hematology, The First Affiliated Hospital of Wenzhou Medical University, Nan Bai Xiang Street, Ouhai District, Wenzhou, Zhejiang 325002 China

**Keywords:** Gastrointestinal, Acute graft-versus-host-disease, Plasma protein, Vascular non-inflammatory molecule 3, Mice

## Abstract

**Background:**

Gastrointestinal acute graft-versus-host disease (GI aGVHD) is a lethal complication following allogeneic hematopoietic stem cell transplantation (HSCT). However, it is still very difficult to make a diagnosis of GI aGVHD in practice. To date, no consensus plasma biomarker of GI aGVHD can be used to help make a diagnosis. Here, we attempted to identify GI aGVHD associated plasma proteins in murine model, which can help make a diagnosis of GI aGVHD.

**Methods:**

We used 8-plex isobaric tags for relative and absolute quantitation (8-plex iTRAQ) to screen out proteins in plasma samples taken from murine models before and after allogeneic HSCT. Next mRNA expressions were validated by quantitative real-time polymerase chain reaction in mouse intestinal epithelial samples.

**Results:**

We found that five proteins were increased at least 2-fold in the allogeneic group at day 7 compared with days 0, 3 and 15 (after Cyclosporin A treatment) and the syngeneic group at day 7. These 5 proteins were VNN3, ZNF746, C4BP, KNG1 and FETUB, and they were consistent with results from negative labeling with 8-plex iTRAQ. Furthermore, increase in mRNA level of VNN3 was confirmed in murine intestinal epithelial samples with aGVHD.

**Conclusions:**

Our results demonstrate that plasma VNN3 protein is associated with GI aGVHD in murine model.

**Electronic supplementary material:**

The online version of this article (10.1186/s12950-017-0178-z) contains supplementary material, which is available to authorized users.

## Background

Acute graft-versus-host-disease (aGVHD), a lethal complication after allogeneic hematopoietic stem cell transplantation (allo HSCT), contributes to high non-relapse mortality [1]. The gastrointestinal (GI) tract is one of the most susceptible organs to aGVHD, and isolated GI involvement is under consideration by researchers [2]. A diagnosis of GI aGVHD is traditionally based on clinical manifestations and endoscopic biopsy [3]. Manifestations of GI aGVHD is characterized by watery diarrhea, nausea, anorexia and abdominal cramps, but these non-specific symptoms may mimic infection [3, 4]. Digestive endoscopy is often restrained by poor general patient health after HSCT, and crypt apoptosis in a mucosal biopsy is not specific for GI aGVHD. [4] Cytomegaloviral (CMV) infection, immunosuppressive medications and other comorbidities may resemble aGVHD histologically [5, 6], complicating the diagnosis of GI aGVHD versus other comorbidities [7].

A serological protein test would be convenient for diagnosis and valuable for predicting clinical outcomes. McDonald’s group used several proteins to predict severe GI aGVHD and non-relapse mortality, such as T-cell immunoglobulin and mucin-domain containing-3, soluble tumor necrosis factor-alpha, interleukin 1 receptor-like 1 and interleukin −6 [8]. Additionally, James and colleagues discovered that regenerating islet-derived 3-alpha in the peripheral blood of patients after allo HSCT as an optional biomarker for diagnosing GI aGVHD before onset [9]. Thus, variations in GI GVHD biomarkers have been reported by different institutions.

Recently, 8-plex isobaric tags for relative and absolute proteomic quantitation (8-plex iTRAQ) has enabled us to identify several biomarker candidates differentially expressed in GI aGVHD patients [10]. However, lots of confounding factors exist in patients’ samples. Thus, we used the mouse models for screening plasma biomarkers of aGVHD to reduce confounding factors. In present study, we used 8-plex iTRAQ proteomic tools to screen out differentially expressed plasma proteins in mouse aGVHD models and identified 5 potential biomarkers: vascular non-inflammatory molecule 3 (VNN3), zinc finger protein 746 (ZNF746), C4b-binding protein (C4BP), kininogen-1 (KNG1) and fetuin (FETUB). Furthermore, these 5 mRNA expressions were tested with real-time polymerase chain reaction (Real-Time PCR) in murine intestinal epithelial samples and VNN3 mRNA expression was validated for GI GVHD. However, confirmation in clinical patient plasma samples for VNN3 is needed.

## Methods

### Mouse model

Male BALB/C (H-2d) and C57BL/6 J (H-2b) mice were purchased from the Chinese Academy of Medical Sciences (HFK, Beijing, China) and housed in groups of five in individually specific pathogen-free barrier facilities. During procedures, animals were kept under a laminar flow hood. Animal diet was standardized pellet chow and UV decontaminated water. All animal work was performed under approved ethical guidelines.

### Bone marrow transplantation (BMT)

Recipient mice (BALB/C, 8 to 11 weeks-of-age) received 8 Gy of total body irradiation (^137^Cs) delivered in two fractions separated by 4 h to reduce GI toxicity. One day afterward, recipients were treated with injected 1 × 10^7^ BM cells (iv) supplemented with 3 × 10^7^ donor spleen cells from age-matched allogeneic donor C57BL/6 J or syngeneic donor BALB/C mice. The allogeneic group consisted of 9 mice undergoing allo-HSCT, and the auto group included 12 mice undergoing auto HSCT.

### Assessment of aGVHD

GVHD severity was assessed with a clinical GVHD scoring system [11] every 2 days and included weight loss, posture, activity, fur texture and skin integrity. At the time of analysis, mice from coded cages were evaluated and graded from 0 to 2 for each criterion. A clinical index was subsequently generated by summation of the five criteria scores (maximum index = 10). As for GI aGVHD, we added diarrhea to these five criteria for diagnosis. Survival was monitored on a daily basis.

### Monitor hematopoietic reconstitution

Blood tests were carried out 3 times a week and evaluation of hematopoietic reconstruction, weight, leukocyte counts exceeding 0.5 × 10^9^/L, or the presence of donor-derived cells was done. Peripheral blood (PB) cells from mice in aGVHD and control groups were stained with PE anti-H-2d and PE-Cy5.5 anti-H-2b (eBioscience, San Diego, CA) as described [12]. FACS CantoII (BD Biosciences, San Jose, CA) were used to acquire data which were analyzed with BD FACSDiva software (BD Biosciences, San Jose, CA).

### Histopathology

Small intestine biopsies were taken from mice at various time points after transplantation. Tissues were placed in 10% buffered formalin phosphate and fixed tissues were paraffin-embedded, sectioned, and stained with H&E. Slides were coded without reference to prior treatment and examined in a blinded fashion by two pathologists.

### CsA, LPSs and PBSs administration

CsA (Cyclosporin A, Novartis PharmaSchweiz AG, Switzerland, 20 mg/kg) was intraperitoneally (ip) administered daily to aGVHD mice. LPS (lipopolysaccharide from *E. coli*, Sigma, MO, USA) was administered (ip, 5 μg) to two auto-graft recipients at day 11 post-transplantation. Phosphate-Buffered Saline (PBS, Sigma, MO, USA) was administered (ip) daily to two auto-graft recipients at day 11 post-transplantation.

### Samples preparation

Plasma and intestinal biopsy specimens were collected from mice using a random approach (allogeneic and syngeneic groups) after animals were cervical dislocated at days 0 (after conditioning and prior to transplantation), 3, and 7 (allogeneic group developed aGVHD and CsA was given, ip), day 11 (syngeneic group PBS or LPS, ip), day 15 (CsA was discontinued in the allogeneic group), day 22 (CsA was given for mice with relapsed aGVHD in the allogeneic group), and day 30.

### Removal of high-abundance proteins

A Seppro IgY-M7 antibody SpinColumn (Sigma, MO, USA) was used to remove the 7 most highly abundant proteins in murine plasma, including albumin, IgG, fibrinogen, transferring, IgM, haptoglobin, and α-1 antitryptase. According to instructions, the low abundant protein samples were prepared from plasma samples.

### Protein concentration assay

Plasma protein was measured using a BCA Protein Assay Kit. After quantification, each sample containing 40 μg protein was preserved at −80 °C.

### Tryptic digestion and iTRAQ labeling

Plasma sample containing 20 μg protein were denatured with buffer 1 (8 M carbamide +100 mM Tris-HCL), and to this was added 4 μl reducing reagent (100 mM dithiothreitol, Amresco, OH, USA) at 37 °C for 2 h, alkylated with freshly prepared cysteine-blocking reagent (500 mM iodacetamide, Amresco, OH, USA) at room temperature for 15 min, and the protein solution was centrifuged at 12,000 rpm for 20 min and the solution at the bottom was collected. Then, to this was added 100 μl buffer 2 (500 mM triethylammonium bicarbonate buffer, Sigma, MO, USA), centrifuged at 12,000 for 10 min, supernatant was discarded and solution at the bottom was collected. This was repeated three times. Subsequently, protein was incubated with 40 μl sequencing grade modified trypsin (Promega, WI, USA) at 50 ng/μl and 100 μl buffer 2 at 37 °C for 16 h.

Digested samples were labeled with iTRAQ reagents (SCIEX, MA, USA) twice (positive and negative). Briefly, one vial of 8-plex iTRAQ labeling reagent (i.e., m/z 113, 114, 115, 116, 117, 118, 119, 121) was used for every 100 μg protein. Positive labeling and negative labeling were shown in Additional file 2. First, iTRAQ reagent was solubilized in 70 μl of isopropanol and then to this was added to 100 μg protein sample for 2 h. Next, labeling was terminated with 100 μl of water. Then, 1 μl of sample was withdrawn from every protein sample and desalinated with Ziptip and identified by MALDI-TOF/TOF to assure labeling was successful. After identification, samples were then mixed at equal ratios and dried with vacuum freeze-drying.

### Protein separation and identification

Labeled proteins were separated with high pH reversed phase chromatography using a C18 trapping column (ZORBAX Extend C18, 100 Å2.1 × 150 mm) at a flow rate of 0.3 ml/min and the wavelengths used were 215 and 280 nm. Lyophilized proteins were dissolved in Nano-RPLC buffer A (2% acetonitrile/water containing 0.1% formic acid), desalted onto a C18 precolumn (100 μm × 3 cm, C18, 3 μm, 150 Å) for 10 min at 2 μL/min, and analyzed on EksigentnanoLC-Ultra™2D system (SCIEX, Beijing, China). Subsequently, the protein were analyzed on a nano-reverse-phase LC system (RPLC), using a C18 reversed phase chromatograph column (75 μm × 15 cm C18- 3 μm 120 Å, ChromXPEksigent), and Nano-RPLC buffer B (98% acetonitrile/water containing 0.1% formic acid) was increased from 5~38% in 70 min. Data were calculated using Protein Pilot Software v. 4.5 (SCIEX, MA, USA).

### Validation in intestinal epithelial samples by quantitative real-time PCR

Total RNA from mice recipients in different groups at different interval times were extracted by TRIzol (Invitrogen, Carlsbad, CA, USA). RNA concentration and quality were quantified by measuring the absorbance at 260 nm with Thermo Scientific™ NanoDrop™ spectrophotometer (Thermo Scientific, Wilmington, DE, USA) and gel analysis. Relative expression was calculated using the 2^-△△CT^ method. The primers of VNN3 and other gene transcripts were designed by Primer Express Software v2.0. β- actin housekeeping gene was used for normalization.

### Reproducibility

Experiments were repeated at least 2 times with 3 sample replicates; To analyze one variable in one BMT experiment, at least two groups of 5 recipients are required.

### Statistical analysis

Bars and error bars represent the means and standard errors of the mean, respectively. An ANOVA test was used to evaluate the difference between each two different groups. A *p* value < 0.05 or 0.001 was considered to be statistically significant. Statistical analyses were performed using GraphPad Prism software (Irvine, CA).

## Results

### Construction of GI aGVHD mouse model

**Fig. 1:**
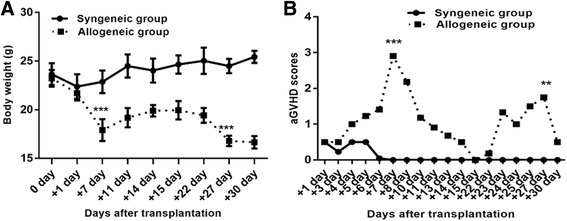
aGVHD was induced in recipient mouse with allogeneic HSCT compared with syngeneic HSCT. **a** Weight changes of recipient mice at different time-points. **b** GVHD scores evaluated in allogeneic and syngeneic groups at different time-points after transplant. **P < 0.05; ***P < 0.001. Bars and error bars represent the means and standard errors of the mean, respectively. *n* = 5 mice per time point per group

**Fig. 2:**
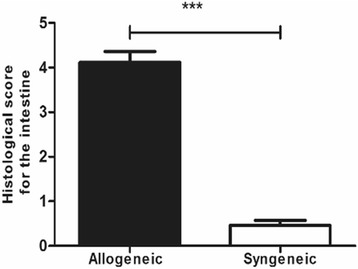
aGVHD was verified by histological changes in allogeneic recipients. Histological scoring analysis of intestinal wall of different groups. ***P < 0.001, *n* = 5 mice per time point per group

HSCs from donor mice were transplanted into irradiated recipient mice as described and mice were divided into allogeneic and syngeneic groups. Seven days after transplantation, mice in the allogeneic group had characteristic aGVHD, including hair loss, skin lesions, hunched postures, diarrhea and weight loss. Significant differences in bodyweight and aGVHD scores relative to syngeneic mice were observed from day 4 onward. The bodyweights of mice in the allogeneic group were significantly lower than those in the control group at day 7 (*P* < 0.05). Similarly, the aGVHD scores of allogeneic transplantation mice differed from the scores of the syngeneic transplantation mice; the gap between the aGVHD scores in the allogeneic and syngeneic groups was largest at day 7 (*P* < 0.001) (Fig. 1a and b). WBC and Neutrophil counts at days 6 and 22 were significantly elevated compared with that at other time points in allogeneic group. Especially at day 6, WBC and Neutrophil counts reached the peak (Fig. 4a and b). Flow cytometry data (Additional file 1) showed nearly 100% of PB cells from mice in the allogeneic group were donor-derived at day 7, which confirmed successful reconstruction of hematopoiesis with donor HSCs. Additionally, histological examinations of intestines demonstrated lymphocyte infiltration in the aGVHD group (day 7) but not in the control group (Additional file 1) and histological scoring analysis revealed different impairment patterns of intestine (Fig. 2) [13, 14]. Then, CsA was administered (ip) daily to aGVHD mice until the allogeneic group was in remission at day 15. The aGVHD relapsed at day 22, and mice received CsA and went into remission again. In contrast, the syngeneic group had a significantly lower aGVHD score than the allogeneic group during the entire observation period of 30 days (Fig. 1b). Mice that received LPS or PBS had no aGVHD symptoms. Thus, we successfully constructed the GI aGVHD murine models after allogeneic HSCT.

### Differential expression of proteins in GI aGVHD identified by 8-plex iTRAQ labeling and MALDI-TOF/TOF

We used 8-plex iTRAQ to identify candidate biomarkers in murine plasma samples taken before and after the onset of aGVHD. We identified and quantified 712 proteins of which 5 were increased at least 2-fold in the allogeneic group at day 7 compared with days 0, 3 and 15 (after CSA treatment) and the syngeneic group at day 7 (Additional file 2). These 5 proteins were VNN3, ZNF746, C4BP, KNG1 and FETUB, and they were consistent with results from negative labeling with 8-plex iTRAQ (Additional file 2).

These five plasma proteins in the allogeneic group increased at the onset of GI aGVHD (day 7), and decreased with remission of GI GVHD after CSA treatment. Furthermore, proteins peaked at day 7, whereas aGVHD symptoms were the most evident at day 7. Compared to the allogeneic group, the five plasma proteins in the syngeneic group did not change appreciably.

In addition, LPS was given to the auto group at day 11 to simulate infectious intestinal milieus. Similar protein was observed in LPS treated and PBS control mice. Protein in the allogeneic group at day 7 during GI aGVHD was greater than that in the syngeneic group treated with LPS at day 11. Thus, these five elevated proteins may not be caused by pathogens such as LPS.

### Validation by real-time PCR in intestinal epithelial samples

**Fig. 3:**
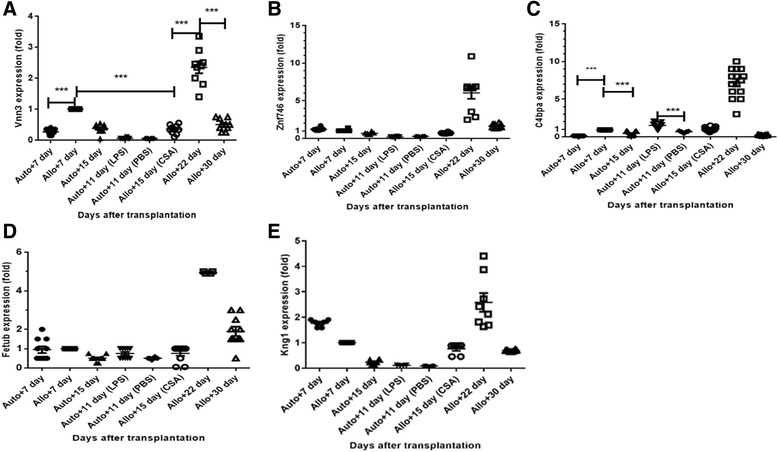
Kinetic fold changes in VNN3, ZNF746, C4BP, KNG1 and FETUB mRNA expression levels in intestinal epithelial cells after HSCT. **a** Kinetic fold changes in VNN3 mRNA expression in intestinal epithelial cells were consistent with protein expression in plasma. VNN3 mRNA expression in the intestinal epithelial cells in the allogeneic group peaked at the onset of GI aGVHD (day 7), and decreased during remission (day 15). Then, it increased again and peaked at day 22 when GI aGVHD relapsed. Expression at day 22 exceeded 6 times the expression at day7. Compared to the allogeneic group, VNN3 mRNA expression in the syngeneic group didn’t change significantly (*P* > 0.05). (**a-e**) VNN3, ZNF746, C4BP, KNG1 and FETUB mRNA expression peaked at day 22 when GI aGVHD recurred. Expression at day 22 was three times the expression at day 7.; ***P < 0.001. mRNA expression fold change at different time points after transplantation are normalized with those at day 7 in the allogeneic group. *n* = 5 mice per time point per group, two sites of intestine in each mouse was tested

To determine whether the five plasma proteins of interest agreed with gene expression data in intestinal epithelial cells, we obtained intestinal epithelial specimens to measure VNN3, ZNF746, C4BP, KNG1 and FETUB gene expression using Real-Time PCR. Kinetic changes in VNN3 expression in the intestinal epithelial cells were consistent with protein expression in plasma (Fig. 3a). [15] According to the results of Real-Time PCR, the VNN3 level of allogeneic group fluctuated as the occurrence and remission of GI aGVHD as well. It has a 2.7-fold increase at the time of aGVHD at day 7 in allogeneic group as compared with corresponding time point of control syngeneic group (*p* < 0.001). After CSA treatment at day 15 of allogeneic group, the VNN3 level decreased 1.8-fold (p < 0.001).When the GI aGVHD relapsed at day 22, the level of VNN3 increased to 2.6 times as against day 15 (*p* < 0.001). By contraries, the VNN3 level of syngeneic group at day 11(after LPS injection) was merely 0.26 times as against day 7 in auto-group (*p* > 0.05). Also, PBS could not cause significant increase of VNN3 level (0.2-fold) as compared with day 7 in syngeneic group (*p* > 0.05).

**Fig. 4:**
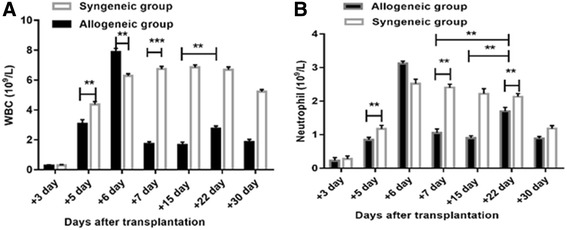
WBC and neutrophil count changes after transplantation in mice. By day 7, recipients in the allogeneic group developed leukopenia, with a 5.5-fold reduction in the WBC count (P < 0.001) (**a**) and a 3.0-fold reduction in the neutrophil count (*P* < 0.05) (**b**) compared with the syngeneic group. At day 22 in allogeneic group, the WBC and neutrophil count were recovered up to about 1.7-fold and 1.9-fold, respectively, in comparison with those in day 15 (*P* < 0.05). **P < 0.05; ***P < 0.001. Bars and error bars represent the means and standard errors of the mean, respectively. *n* = 5 mice per time point per group

Besides, the expression of gene VNN3, ZNF746, C4BP, KNG1 and FETUB achieved the peak at day 22 when the GI aGVHD recurred and were more than 3 times as those at day 7(Fig. 3a-e). In our study, the count of WBC especially neutrophil at day 22 is much more than that at day 7 as well (Fig. 4a and b). However, kinetic changes of RNA ZNF746, C4BP, KNG1 and FETUB levels were not consistent with those changes in plasma (Fig. 3b-e).

## Discussion

The complicated pathophysiology of GI aGVHD is accompanied by breaches in the GI mucosa and pathogen transduction, allo-reactivity of T cells, inflammation, and tissue damage and repair [3, 16–18]. We report here that five differential expression of plasma proteins VNN3, ZNF746, C4BP, KNG1 and FETUB between aGVHD+ and aGVHD- plasma samples in mouse model. All five plasma proteins VNN3, ZNF746, C4BP, KNG1 and FETUB have not been described in GI aGVHD in previous studies. These five plasma proteins were screened by relative and absolute quantitation proteomic technology (8-plex iTRAQ), which can increase analytical throughput while reducing experimental error and improve peptide ionization [10, 19]. Nevertheless, few published studies suggested iTRAQ proteomic tools for searching aGVHD biomarkers in plasma samples. Furthermore, VNN3 gene expression was validated in intestinal epithelial samples by Real-Time PCR (Fig. 3a). Thus, elevated plasma VNN3 may be produced by damaged GI epithelial cells when aGVHD occurred.

VNN3, a Vanin family member encoding pantetheinase isoforms, hydrolyzes pantetheine into pantothenic acid (vitamin B5) and cysteamine, which is an antioxidant [20, 21]. In vivo, pantothenic acid requires transformation into coenzyme-A to metabolize proteins, carbohydrates and fats. Nitto’s group found alternative spliced variants of VNN3 in leukocytes lysates, especially in neutrophils [22]. Also, VNN3 is an ectoenzyme and can be secreted by neutrophils [21–23], which cause inflammation in transplanted organs. Recently, neutrophils have been reported to have analogous function to antigen presenting to lymph nodes and promoting differentiation of T cells, such as APCs [24, 25].

Of note, in our study, neutrophil counts reached the peak at day 6 upon the fulminant aGVHD and reached the second peak at day 22 with the relapsed aGHVD (Fig. 4b). The initial breaches to the GI mucosal barrier may be caused by the conditioning regimen allows translocation of potent inflammatory stimuli such as LPS, high Mobility Group Box-1 (HMGB-1) and other pathogen-associated molecular patterns [3, 26–28], which bind to Toll-like receptor in neutrophils and trigger activation and production of ectoenzyme [3, 26, 27]. To “tackle” the inflammatory cascade upstream, in some cases, LPS and HMGB-1 could be eliminated by AN69 membranes in dialysis [29].

Therefore, VNN3 increases in PB and its products pantothenic acid and cysteamine increase subsequently to provide the requisite energy and anti-oxidation with oxidative status of neutrophils [16, 30]. Thus, at the onset of GI aGVHD, expression of VNN3 in intestinal epithelial cells increased and as the intestinal epithelium was damaged, intracellular synthetic VNN3 protein was released and increased in the plasma, which may provide protection effect. In addition, VNN3 expression was decreased after CSA treatment in our study, which indicates VNN3 plasma protein may have value not only as a noninvasive diagnostic biomarker but also as an evaluative index for treatment effect. Furthermore, VNN3 expression was not increased after LPS administration, which may be helpful for differentiation of aGVHD from infection.

Although the kinetics of the other 4 RNAs (ZNF746, C4BP, KNG1 and FETUB) expression in intestinal epithelial cells in our Real-Time PCR test are not coordinated with plasma changes, these proteins had concordant changes between positive and negative 8-plex iTRAQ labelling. Hence, these four plasma proteins may be also involved in the “cytokine storm” of aGVHD. ZNF746, a substrate of parkin that plays a role in innate immune defense mechanisms, [31] may assist in preventing GI aGVHD which can be triggered by innate immunity [3, 26, 27, 32]. Because of the consumption of parkin in GI aGVHD, ZNF746 can be upregulated. C4BP inhibits activation of the complement cascade, which offers anti-inflammatory activity in GI aGVHD with complex pro-inflammatory milieus [33]. FETUB is implicated in the production of TNF-α and IL-6 that are key to the pathophysiology of GI aGVHD [34–36]. KNG1 was reported to contribute to experimental intestinal and systemic inflammation and high KNG1 may damage tissue and may be upregulated in GI aGVHD [37].

As shown in Fig. 3a-e, gene expression of VNN3, ZNF746, C4BP, KNG1 and FETUB peaked at day 22. Meanwhile, neutrophils counts at day 22 also markedly exceeded that on day 7 (Fig. 4b, *p*<0.05). Accordingly, neutrophils might cause much more inflammation in GI at day 22 that other time points. Several studies indicate that neutrophils proliferate and migrate to the GI tract where they cause tissue damage and promote the development of GI aGVHD [38–41]. Neutrophils, cleaving chemokines and producing reactive oxygen species, promote activation of T cells and consequently aggravate GI aGVHD [42]. A detailed pathologic study indicated that greater neutrophils density in the full thickness of the lamina propria were associated with worse clinical outcome [39]. All of these studies strongly suggest that neutrophils are correlated with the severity of inflammation damage.

## Conclusions

Plasma VNN3 protein is associated with GI aGVHD in mouse models. In addition, matching differential expression of plasma protein with corresponding gene expression in intestinal epithelium indicates the high likelihood of replacing intestinal biopsy with noninvasive plasma biomarker. In future studies, we will validate VNN3 with ELISA in patient plasma samples to determine their significance with respect to clinical outcome.

## Additional files

Additional file 1: aGVHD was verified by histological changes in allogeneic recipients. (A and B) Donor-derived cells in the allogeneic group were measured by flow cytometry at day 7 to confirm implantation of donor. (C) H&E staining of mouse small intestine tissues in the allogeneic group on day 7. Small intestinal mucous villi degenerate and denude. (D) H&E staining of mouse small intestinal tissues of mice in the syngeneic group on day 7. Small intestinal mucosa had no obvious pathological changes. *n*=5 mice per time point per group. (DOC 3783 kb)

Additional file 2: Positive iTRAQ label of proteins with increased levels in day 7 in allogeneic group with aGVHD.8-plex iTRAQ approach is that the relative quantification is achieved via the difference in abundance of the reporter product ions (i.e., m/z 113, 114, 115, 116, 117, 118, 119, 121). For positive labeling, 115 was day 0, 114 was day 3 (allogeneic group), 116 was day 7 (syngeneic group), 117 was day 7 (allogeneic group), 118 was day 11(syngeneic group ip PBS), 119 was day 11 (syngeneic group ip LPS), and 121 was day 15 (after CsA treatment in allogeneic group), 113 was labeled day 22 (syngeneic group). Differential protein levels between aGVHD+ and aGVHD- from eight time points were evaluated for 117/115, 117/114, 117/116, 117/121, 117/118, 117/119 and 117/113 sets, respectively, with the ratio more than 2.0 defined as increased. (DOC 48 kb)

## Abbreviations

aGVHD: Acute graft-versus-host-disease
allo-HSCT: Allogeneic hematopoietic stem cell transplantation
C4BP: C4b-binding protein
CMV: Cytomegaloviral
CsA: Cyclosporin A
FETUB: Fetuin
GI: Gastrointestinal
Ip: Intraperitoneally
iTRAQ: Isobaric tags for relative and absolute quantitation
KNG1: Kininogen-1
LPS: Lipopolysaccharide
PBS: Phosphate-buffered saline
PCR: polymerase chain reaction
VNN3: Vascular non-inflammatory molecule 3
ZNF746: Zinc finger protein 746
