# Supplementary material for: Plasma vascular non-inflammatory molecule 3 is associated with gastrointestinal acute graft-versus-host disease in mice
Source: J Inflamm (Lond). 2018 Jan 5;15:1. doi: 10.1186/s12950-017-0178-z (PMC5755465; doi:10.1186/s12950-017-0178-z)
Supplement: Supplementary file 1 — aGVHD was verified by histological changes in allogeneic recipients. (A and B) Donor-derived cells in the allogeneic group were measured by flow cytometry at day 7 to confirm implantation of donor. (C) H&E staining of mouse small intestine tissues in the allogeneic group on day 7. Small intestinal mucous villi degenerate and denude. (D) H&E staining of mouse small intestinal tissues of mice in the syngeneic group on day 7. Small intestinal mucosa had no obvious pathological changes. n=5 mice per time point per group. (DOC 3783 kb) [file 12950_2017_178_MOESM1_ESM.doc]

**Fig. 1**


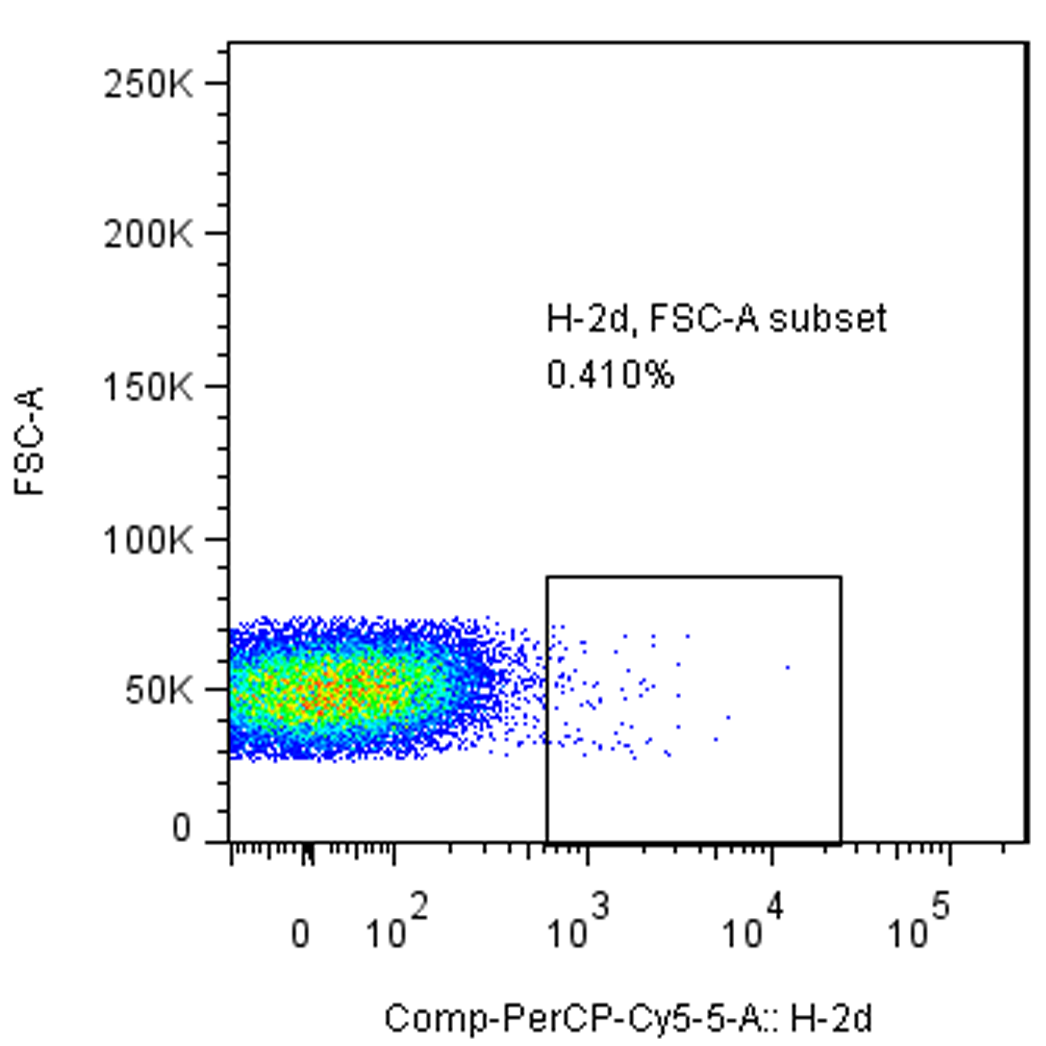

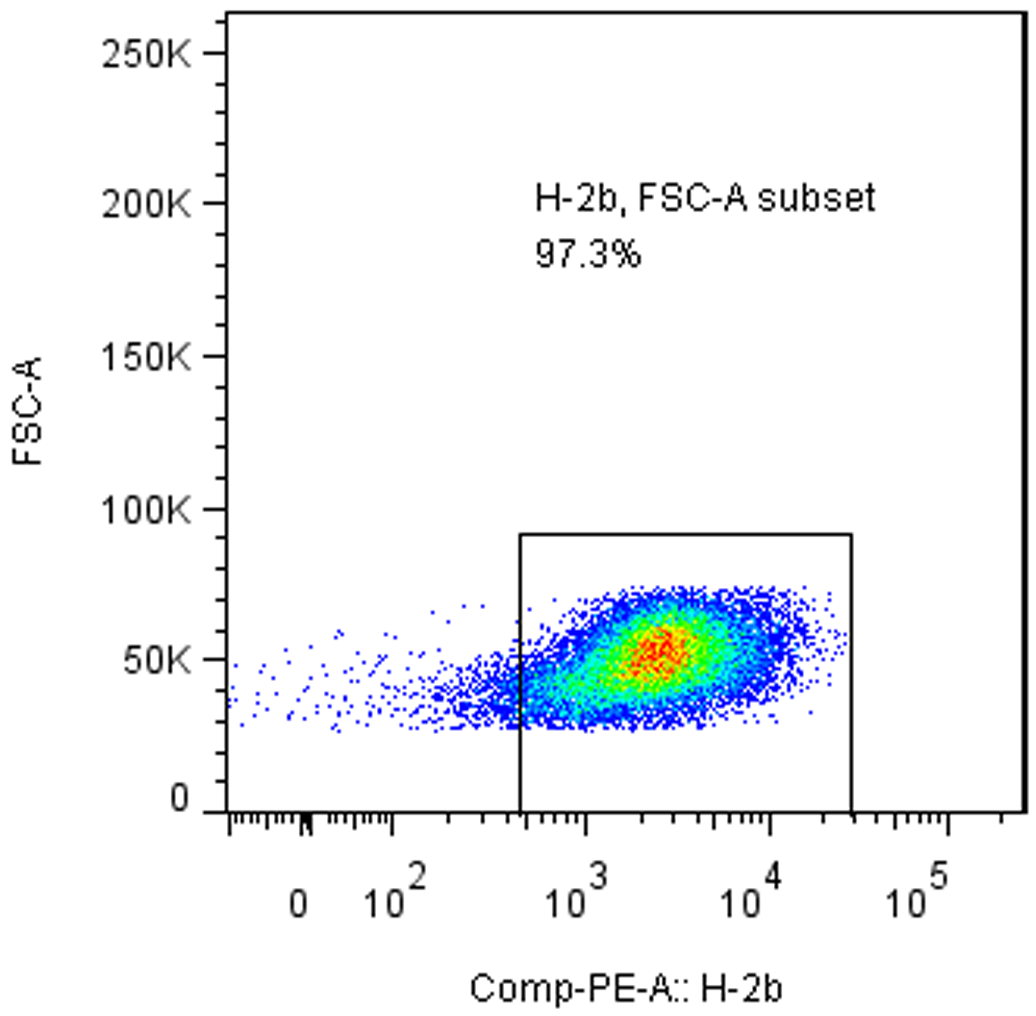

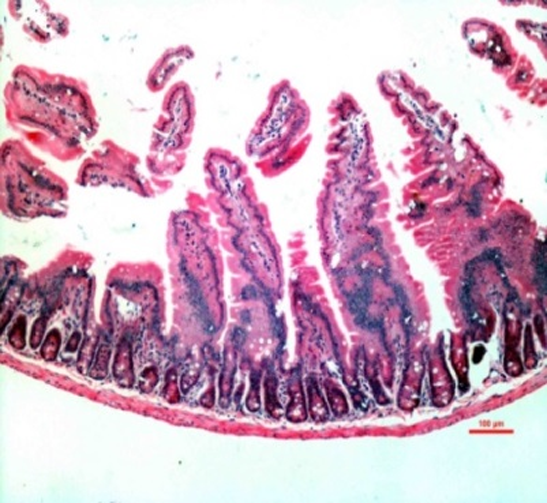

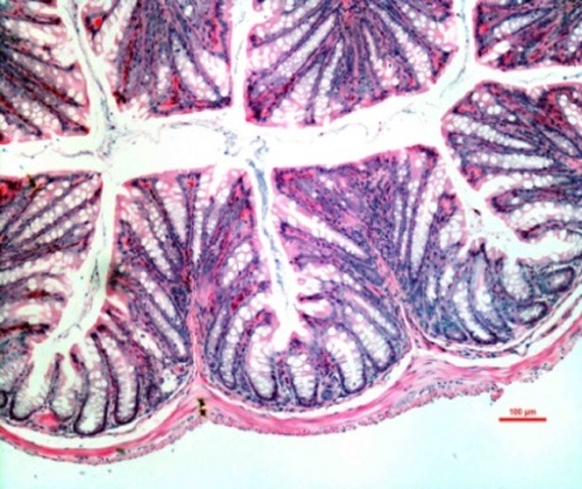


Additional Fig.1A

Additional Fig.1B

Additional Fig.1C

Additional Fig.1D

**Fig. 1.** aGVHD was verified by histological changes in allogeneic recipients. (A and B) Donor-derived cells in the allogeneic group were measured by flow cytometry at day 7 to confirm implantation of donor. (C) H&E staining of mouse small intestine tissues in the allogeneic group on day 7. Small intestinal mucous villi degenerate and denude. (D) H&E staining of mouse small intestinal tissues of mice in the syngeneic group on day 7. Small intestinal mucosa had no obvious pathological changes. n=5 mice per time point per group.
