# Supplementary material for: Plasma vascular non-inflammatory molecule 3 is associated with gastrointestinal acute graft-versus-host disease in mice
Source: J Inflamm (Lond). 2018 Jan 5;15:1. doi: 10.1186/s12950-017-0178-z (PMC5755465; doi:10.1186/s12950-017-0178-z)
Supplement: Supplementary file 2 — Positive iTRAQ label of proteins with increased levels in day 7 in allogeneic group with aGVHD.8-plex iTRAQ approach is that the relative quantification is achieved via the difference in abundance of the reporter product ions (i.e., m/z 113, 114, 115, 116, 117, 118, 119, 121). For positive labeling, 115 was day 0, 114 was day 3 (allogeneic group), 116 was day 7 (syngeneic group), 117 was day 7 (allogeneic group), 118 was day 11(syngeneic group ip PBS), 119 was day 11 (syngeneic group ip LPS), and 121 was day 15 (after CsA treatment in allogeneic group), 113 was labeled day 22 (syngeneic group). Differential protein levels between aGVHD+ and aGVHD- from eight time points were evaluated for 117/115, 117/114, 117/116, 117/121, 117/118, 117/119 and 117/113 sets, respectively, with the ratio more than 2.0 defined as increased. (DOC 48 kb) [file 12950_2017_178_MOESM2_ESM.doc]

Table 1 Positive iTRAQ label of proteins with increased levels in day 7 in allogeneic group with aGVHD

| 117/115 117/114 117/116 117/121 117/118 117/119 117/113 |
| --- |
| Vascular non-inflammatory molecule 3 9.8 15.3 11.3 23.8 13.0 19.2 4.7  Kininogen-1 6.0 6.4 9.0 4.6 6.1 3.9 6.9  Fetuin-B 2.8 4.7 2.6 2.1 3.6 2.0 2.0  C4b-binding protein 3.0 10.4 12.8 8.3 6.4 5.1 10.8  Zinc finger protein 746 2.9 4.4 5.8 2.4 4.8 2.7 4.0 |

8-plex iTRAQ approach is that the relative quantification is achieved via the difference in abundance of the reporter product ions (i.e., m/z 113, 114, 115, 116, 117, 118, 119, 121). For positive labeling, 115 was day 0, 114 was day 3 (allogeneic group), 116 was day 7 (syngeneic group), 117 was day 7 (allogeneic group), 118 was day 11(syngeneic group ip PBS), 119 was day 11 (syngeneic group ip LPS), and 121 was day 15 (after CsA treatment in allogeneic group), 113 was labeled day 22 (syngeneic group). Differential protein levels between aGVHD+ and aGVHD- from eight time points were evaluated for 117/115, 117/114, 117/116, 117/121, 117/118, 117/119 and 117/113 sets, respectively, with the ratio more than 2.0 defined as increased.

Table 2 Negative iTRAQ label of proteins with increased levels in day 7 in allogeneic group with aGVHD

| 116/118 116/119 116/117 116/113 116/115 116/114 116/121 |
| --- |
| Vascular non-inflammatory molecule 3 9.0 9.0 5.4 7.9 7.5 11.2 4.0  Kininogen-1 3.5 4.4 7.0 4.3 6.5 4.8 6.3  Fetuin-B 2.8 5.2 2.8 3.4 6.0 2.3 3.1  C4b-binding protein 2.9 8.9 16.6 13.4 7.4 6.2 5.9  Zinc finger protein 746 3.4 8.2 30.5 2.2 5.1 2.2 20.7 |

For negative labeling, 118 was day 0, 119 was day 3 (allogeneic group), 117 was day 7 (syngeneic group), 116 was day 7 (allogeneic group), 113 was labeled day 15 (after CsA treatment in allogeneic group), 114 was day 11 (syngeneic group ip LPS), 115 was day 11(syngeneic group ip PBS), and 121 was day 22 (syngeneic group). Differential protein levels between aGVHD+ and aGVHD- from eight time points were evaluated for 116/118, 116/119, 116/117, 116/113, 116/115, 116/114 and 116/121 sets, respectively, with the ratio more than 2.0 defined as increased
